# Intraoperative Anesthesia-Related Critical Events in Low-Resource Hospitals During Short-Term Surgical Missions in Tanzania and Democratic Republic of the Congo: An Observational Study

**DOI:** 10.1213/ANE.0000000000007317

**Published:** 2024-10-28

**Authors:** Simon Ponthus, Amina Omari, Selerina Tesha, Castram Mbuza, Alexis Peruzzo, Pierre Kabuya, Richard Yamuremye, Lionel Dumont

**Affiliations:** From the * Department of Anesthesiology, Pharmacology, Intensive Care and Emergency Medicine, University Hospitals of Geneva, Geneva, Switzerland; † 2^nd^ Chance Association, Geneva, Switzerland; ‡ Department of Anesthesiology, Tumbi Regional Referral Hospital, Kibaha, Tanzania; § Department of Anesthesiology, Provincial General Hospital of Goma, Goma, Democratic Republic of Congo; ‖ Faculty of Medicine, University of Geneva, Geneva, Switzerland; ¶ Department of Anesthesiology, Provincial General Hospital of Bukavu, Bukavu, Democratic Republic of Congo; # Department of Anesthesiology, Centre Hospitalier Universitaire Kamenge Bujumbura, Bujumbura, Burundi.

## Abstract

**BACKGROUND:**

Critical incidents and mortality related to anesthesia are more frequently observed in low- and middle-income countries in comparison to high-income countries. The difficulties linked to anesthesia in rural areas of the Democratic Republic of the Congo (DRC) and Tanzania have limited documentation. The aim of this study was to comprehensively document anesthesia-related critical events that occurred during surgical missions organized by the nongovernmental organization 2^nd^ Chance in hospitals in DRC and Tanzania.

**METHODS:**

Data were collected during 6 surgical missions in 3 hospitals in the DRC and 1 in Tanzania. All scheduled surgery patients were included. Anesthesia was administered by a local Non-Physician Anesthesia Provider (NPAP), using local resources, under the supervision of an anesthesiologist from the association. The anesthesiologist reported critical events and collected data. Local teams managed critical events initially, with intervention by the anesthesiologist from 2^nd^ Chance on the local team’s request, according to preestablished protocol or if the situation was considered dangerous. Critical incidents associated with anesthesia, including bradycardia, hypoxemia, airway management failure, and equipment problems, were documented from induction of anesthesia until discharge from the recovery room.

**RESULTS:**

We recruited 201 patients, of whom 192 were evaluated, with 9 patients dropping out due to protocol noncompliance. All patients were American Society of Anesthesiologists (ASA) I (62%; n = 120) or ASA II (38%; n = 72). Among them, 104 individuals (54%) experienced at least 1 critical event, totaling 202 critical events. Hypoxemia emerged as the most common event, affecting 29% of the patients (n = 55) with at least 1 episode. Equipment problems (oxygen supply and/or anesthesia machine failure) occurred in 24% of cases (n = 46), airway management issues in 23% (n = 44), and bradycardia in 6% (n = 12). Hypotension and hypertension were not documented due to the lack of monitoring. The majority of these events (over 60%) required intervention by the anesthesiologist.

**CONCLUSIONS:**

The occurrence of critical events related to anesthesia appears to be high in this study. Due to numerous limitations, these results cannot be generalized to all hospitals in Tanzania and the DRC. However, this study underscores the challenges faced by anesthesia teams, encompassing inadequate resources, equipment deficiencies, and varying levels of expertise among anesthesia personnel. The research further stresses the significance of addressing these challenges to enhance patient safety.

**KEY POINTS**

**Question:** What is the incidence of anesthesia-related critical events in low-resource hospitals in Tanzania and in the Democratic Republic of the Congo during short-term missions of reconstructive and giant goiter surgery?

**Finding:** In this study, performed with a specific design during surgical missions, the incidence of anesthesia-related critical events was 54%.**Meaning:** Incidence of anesthesia-related critical events is high in this study and may be explained by a variety of factors.

Anesthesia-related serious incidents and mortality are more prevalent in low- and middle-income countries compared to high-income countries.^1,2^ The available data on anesthesia-related critical events in resource-limited countries mainly consist of epidemiological data^3,4^ or results from meta-analyses,^2,5^ with only a few case-by-case field studies.^6^ It is now evident that anesthesia becomes more perilous when resources are limited.^1,7^ There is a lack of observational field data capturing experiences of anesthesia providers working with locally available resources and reporting critical events.^6^ Such close-up observations are invaluable for understanding the causes behind anesthesia-related critical incidents. The specific contexts in which these incidents occur are often underreported without comprehensive consideration of the contributing environment.

Very few sub-Saharan African hospitals meet the International Standards of a Safe Practice of Anesthesia as laid out by the World Health Organization (WHO) and World Federation of Societies of Anaesthesiologists (WFSA).^8^ This is particularly true in rural areas.^9–11^ To mitigate these disparities in terms of equipment, the Lifebox initiative has made it possible for virtually all hospitals, to have at least 1 pulse oximeter, which often serves as the only monitoring device.^12,13^

2^nd^ Chance Association is a nonprofit organization that undertakes short-term missions to sub-Saharan Africa to provide surgical services for giant goiters and for reconstructive surgery (mostly postburn contracture).^14^ 2^nd^ Chance anesthesiologists work with local Non-Physician Anesthesia Providers (NPAPs) to provide anesthesia, using local resources only, except for the capnographs. An experienced visiting anesthesiologist is paired with a local NPAP. They engage in a collaborative exchange. This unique design ensures safety, even in situations with limited resources. Over the last 10 years, the surgical missions have reported no perioperative mortality.^14^ This success underscores the effectiveness of this approach in promoting safe anesthesia practices within resource-constrained settings. NPAPs face daily challenges while conducting anesthesia procedures without proper monitoring, drugs, or anesthesia machines. Additionally, they face issues such as having lower professional status than surgeons, challenging responsibilities, inadequate education, and financial acknowledgment from institutions. The challenges related to anesthesia in the DRC and Tanzania are poorly documented and overshadowed by other health-related problems facing the authorities.^15,16^ The aim of 2^nd^ Chance’s short-term surgical missions is to assist Tanzanian and Congolese colleagues to improve safety standards in anesthesia. By documenting recurring safety issues, the goal is to encourage the recognition and safe management of problems and to advocate for the necessary resources.

**Table 1.** Characteristics of the Hospitals Included in the Study

General characteristics															
Name of the hospital	City/area/country			No. of bed		Population covered by the hospital (million)		WHO health care facility level^a^		PACU functional	Number NPAPs				
Tumbi regional referral hospital	Kibaha/Pwani Area/Tanzania			281		1.1		2		0	6				
Nebobongo district hospital	Nebobongo/Haut Uele Area/ DRC			120		0.26		2		0	2				
Hôpital du cinquantenaire	Kisangani/Thsopo Area/ DRC			300		1.6		2		0	2				
Provincial General Hospital of Bukavu	Bukavu/South Kivu Area/ DRC			450		1		3		0	15^b^				
**General characteristic focus on the study**															
**Surgery**		**Population**	**Type of anesthesia**		**Monitoring**							**Main medications**			
					**Always available**		**Sometimes available**		**Anesthesia machine (n functional)**			**Volatile agent**	**Muscle relaxant**	**Opoids**	**Analgesic**
Reconstructive surgery		Adult & Pediatric	GA SV + RA		Oximeter		BP adult + EKG		Manual circuit + sodalime Oxygen tank & oxygen concentrator. MV not available (n = 3)			Halothane	Suxamethonium	Fentanyl	Paracetamol + NSAID+ Tramadol
Giant Goiter		Adult	GA SV		Oximeter		BP manual		Draw over + oxygen tank & oxygen concentrator. (n = 1)			Halothane	Suxamethonium	Fentanyl	Tramadol
Giant Goiter		Adult	GA SV		Oximeter		BP manual		Draw over + oxygen tank & oxygen concentrator. (n = 2)			Halothane	Suxamethonium	Fentanyl	Tramadol
Giant Goiter		Adult	GA SV		Oximeter		BP automatic + EKG		Manual circuit + sodalime Oxygen tank & oxygen concentrator. MC available (n = 4)			Halothane	Suxamethonium	Fentanyl	Paracetamol + NSAID+ Tramadol

Abbreviations: BP, blood pressure; DRC, Democratic Republic of the Congo; EKG, electrocardiogram; GA, general anesthesia; MV, mechanical ventilation; NPAPs, non-physician anesthesia providers; NSAID, nonsteroidal anti-inflammatory drugs; PACU, postanesthesia care unit; RA, regional anesthesia; SV, spontaneous ventilation; WHO, World Health Organization.

a Level 1 = small hospital/ health center; Level 2 = district/ provincial hospital; Level 3 = referral hospital

b Among them, 1 medical doctor specialized in anesthesiology.

**Table 2.** Characteristics of the Patients

Characteristic	All patients (n = 192)
Country	
Tanzania, n(%)	143 (75%)
Democratic Republic of the Congo, n(%)	49 (25%)
Sex (M/F)	74/118
Age	
Mean (SD)	17 (21)
0–1 y	17 (8.85%)
2–5 y	82 (42.71%)
6–21 y	30 (15.63%)
> 21 y	63 (32.81%)
ASA status	
I, n(%)	120 (62%)
II, n(%)	72 (38%)
III, n(%)	0
IV, n(%)	0
Surgical procedure	
Reconstructive surgery, n(%)	143 (75%)
Giant goiter, n(%)	49 (25%)
Anesthesia procedure	
General alone, n(%)	106 (55%)
Combined (general and regional), n(%)	86 (45%)

n indicates total number, and % indicates proportion of total cases (n).

Abbreviation: ASA, American Society of Anesthesiologists; SD, standard deviation.

**Table 3.** Critical Events During General Anesthesia Among the Patients, By Age, By Country, and Total

Subgroups	0–1 y	2–5 y	6–21 y	>21 y	Tanzania	DRC	Total
n patients	17	82	30	63	143	49	192
Patient with at least 1 critical event, n(%)	8 (47%)	43 (52%)	18 (60%)	35 (56%)	73 (51%)	31 (63%)	104 (54%)
Number of critical events (total), n	15	94	27	66	142	60	202
Bradycardia (at least 1), n(%)	2 (12%)	6 (7%)	2 (7%)	2 (3%)	11 (8%)	1 (2%)	12(6%)
Airway management problem (at least 1), n(%)	3 (18%)	20 (24%)	7 (23%)	14 (22%)	31 (22%)	13 (27%)	44 (23%)
Hypoxemia (at least 1), n(%)	4 (24%)	28 (34%)	12 (40%)	11 (17%)	47 (33%)	8 (16%)	55 (29%)
Equipment incident during anesthesia (at least 1), n(%)	5 (29%)	15 (18%)	4 (13%)	22 (35%)	25 (18%)	21 (43%)	46 (24%)

n indicates total number, and % indicates proportion of total cases (n).

Abbreviation: DRC, Democratic Republic of the Congo.

**Table 4.** Hypoxemia Events: Characteristics and Etiology

Patient subgroups	1 event of hypoxemia	2 events of hypoxemia	3 events of hypoxemia	Total
Number of patients	37	10	8	55
Number of hypoxemia events, n	37	20	24	81
Age, mean (SD)	11 (16)	11 (17)	14 (21)	12 (17)
0–1 y (n = 4)/10 (6) months	2	1	1	4
2–5 y (n = 28)/3 (1)	17	6	5	28
6–21 y (n = 12)/ 8 (3)	11	1	0	12
>21 y (n = 11)/ 43 (14)	7	2	2	11
ASA status				
I, n (%)	27 (73%)	6 (60%)	6 (75%)	39 (71%)
II, n (%)	10 (27%)	4 (40%)	2 (25%)	16 (29%)
III, n (%)	0	0	0	0
IV, n (%)	0	0	0	0
Sex (M/F)	19/18	7/3	4/4	30/25
Country				
Tanzania (reconstructive surgery), n (%)	33 (89%)	8 (80%)	6 (75%)	47 (85%)
DRC (giant goiter surgery), n (%)	4 (11%)	2 (20%)	2 (25%)	8 (15%)
Timing of hypoxemia				
Induction, n (%)	10 (27%)	3 (15%)	12 (50%)	25 (30%)
Maintenance, n (%)	8 (22%)	10 (50%)	10 (42%)	28 (35%)
Emergence, n (%)	19 (51%)	7 (35%)	2 (8%)	28 (35%)
Duration of hypoxemia in seconds, mean (SD)	92 (107)	91 (84)	130 (143)	100 (85)
Lowest pulse oximeter value (%), mean (SD)	76 (11)	69 (21)	75 (14)	74 (11)
Minimal pulse oximeter value during hypoxemia	54	35	49	35
Maximal pulse oximeter value during hypoxemia	90	90	90	90
Cause of hypoxemia				
Airway management failure, n (%)	13 (35%)	6 (30%)	6 (25%)	25 (31%)
○ Intubation failure, n	5	3	3	11
○ Endobronchial intubation, n	4	3	2	9
○ Facemask ventilation failure, n	2	0	0	2
○ Accidental extubation, n	2	0	1	3
Bronchospasm, n (%)	5 (13%)	3 (15%)	7 (29%)	15 (19%)
Hypoventilation, n (%)	5 (13%)	3 (15%)	5 (20%)	13 (16%)
Laryngospasm, n (%)	6 (17%)	3 (15%)	0	9 (11%)
Anesthesia machine failure, n (%)	1 (3%)	2 (10%)	3 (13%)	6 (8%)
Oxygen supply failure, n (%)	5 (13%)	2 (10%)	3 (13%)	10 (12%)
Aspiration, n (%)	1 (3%)	0	0	1 (1%)
Other, n (%)	1 (3%)	1 (5%)	0	2 (2%)

n indicates total number, and % indicates proportion of total cases (n).

Abbreviation: ASA, American Society of Anesthesiologists; DRC, Democratic Republic of the Congo; SD, Standard Deviation.

The objective of this study was to report all critical events related to anesthesia that occurred intraoperatively during surgical missions organized by 2^nd^ Chance in resource-limited hospitals. We hypothesized that the incidence of critical events might be substantially higher than in high-income countries (HICs).^3^

## METHODS

This study was a prospective observational investigation of a series of cases. The study received approval from the Ethics Committee of the University of Bukavu (UCB/CIES/NC/01212021), the Ethics Committee of the COSECSA College of Surgeons of Eastern, Central, and Southern Africa (IRB Registration Number: 00011122), and the Ethics Committee of the National Institute for Medical Research of the United Republic of Tanzania (no specific registration number attributed to the project). Before participation, all patients involved in the 2^nd^ Chance programs provided their written informed consent, with the assistance of a translator, for the collection and utilization of their medical data for scientific purposes, as needed.

### Geographic, Logistical, and Organizational Aspects

The study was conducted in 4 different hospitals (3 hospitals in DRC and 1 in Tanzania) during 6 missions, from May 2021 to December 2022 in DRC and from December 2022 to March 2024 in Tanzania (Table 1). These surgical missions spanned 5 days each. All patients scheduled for surgery were included in the observation.

### The Key Players in Anesthesia

The composition and size of anesthesia teams in Tanzanian and Congolese hospitals vary, with the exception of Bukavu, where a well-trained anesthesiologist is present. In other hospitals, the anesthesia team consists of NPAP such as technicians, nurses, or anesthesia officers. Their training backgrounds differ, and some have more experience than others (between 1 and 11 years of experience, mean (SD): 4.5 (3)). In general, NPAP training in Tanzania and the DRC is primarily theoretical and typically spans a period of between 1 and 3 years. The curriculum encompasses instruction in physiology, pharmacology, and an array of anesthesia techniques. However, they all perform both adult and pediatric anesthesia on a daily basis, and are also responsible for night shifts to handle emergencies and obstetric cases. The trainers who supervise the NPAP during the observations are experienced anesthesiologists from Europe, the United States, or Africa, with extensive experience in anesthesia within resource-limited settings.

### Anesthesia Procedure

In eastern DRC, patients underwent extensive goiter surgery. Meanwhile, patients from Tanzania underwent reconstructive surgery, mainly for postburn contracture release. Both types of surgery necessitated the use of general anesthesia. For adults and children older than 8 years of age, the anesthesia protocol consisted of intravenous induction using propofol and, when accessible, fentanyl for pain management during intubation. Subsequently, halothane was used for the remainder of the procedure. Intraoperative analgesia involved a combination of local anesthesia at the surgical site or regional anesthesia such as axillary block and intravenous opioids (such as fentanyl or pethidine) at doses appropriate to maintain spontaneous respiration. Anesthesia was induced in children 0 to 8 years of age using an inhalation technique with Halothane (up to 3%). The carrier gas used was 100% oxygen. An intravenous infusion was started after induction. Suxanethonium 1 to 1.5 mg/kg was given to facilitate intubation. Anesthesia maintenance was then continued with halothane along with local or regional anesthesia techniques. Throughout the procedures, a pulse oximeter was consistently available. Access to electrocardiogram (EKG) was infrequent due to the lack of electrodes. Blood pressure measurement was available sometimes for adults but seldom for children due to the lack of suitable pressure cuff sizes. No oxygen or anesthesia gas analyzers were available. Capnography was not available locally but provided by 2^nd^ Chance and had to be shared between operating rooms and patients.

### Data Source and Measurement

Data from all patients included in the study were collected using a standardized form (Supplemental Digital Content 1, Supplemental Table 1, http://links.lww.com/AA/F105). Each patient was assigned a unique number, which was subsequently removed during analysis to ensure the anonymity of each individual.

### The Course of the Study

Each patient in the study was under the care of a team comprising 1 or 2 local NPAPs and an experienced senior expatriate anesthesiologist (AEXP). Before the start of the procedure, both the NPAP and AEXP reviewed the anesthetic strategy and the patient’s file together. The local NPAP was responsible for preparing the equipment and drugs, which were cross-checked by AEXP. Among the NPAP members, 1 was designated as the anesthesia leader for the patient. The anesthesia leader made decisions regarding the sequence of injections, the drugs used, ventilation, intubation, and tube fixation, remaining present throughout the procedure. If an axillary block was required, another NPAP performed it under the supervision of AEXP. For local anesthesia at the surgical site, the surgeon was in charge. AEXP’s role involved observing and documenting any critical incidents related to anesthesia. If necessary, AEXP would intervene either at the request of the NPAP or if the situation was deemed dangerous and potentially harmful. However, the general principle was to let the local NPAP manage the entire procedure without interference. At the end of the procedure, the NPAP extubated the patients and transferred them to the Post-Anesthesia Care Unit (PACU). In the PACU, another team of nurses, supervised by AEXP, took care of the patient until they were ready for discharge. Patients were included in the study if the procedure was entirely managed by the NPAP, and AEXP only intervened when a situation exceeded the NPAP’s capability to handle it.

### Data Collection

The data collection process involved gathering general characteristics of the patients, such as gender, age, American Society of Anesthesiologists (ASA) status, weight, type of anesthesia, type of surgery, and duration of the surgical procedure. Regarding “anesthesia-related critical events,” we adopted the definition provided by de Graaf,^17^ which has been subsequently used by others.^3,9^ We defined critical events as follows. Hypoxemia was defined as a drop in oxygen saturation below 90% on pulse oximetry, regardless of the duration. Each occurrence was meticulously recorded, providing information on the timing, duration, lowest recorded value, cause of the event, and whether the local team managed it independently or with assistance. In the case of bradycardia, the definition was a heart rate below 50 beats per minute in adults, with age-adjusted criteria for children. Additionally, equipment failure was defined as any malfunction in anesthesia equipment that had the potential to compromise patient safety. Airway management failure was defined as either: facemask ventilation failure, >2 laryngoscopies, endobronchial intubation, esophageal intubation, accidental extubation. The detailed documentation of critical events (data form) can be found in Supplemental Digital Content 1, Supplementary material, http://links.lww.com/AA/F105. To minimize the risk of overestimation, 2 investigators, independently verified the occurrence of critical events. The 2 investigators were the AEXP and another anesthesiologist from 2^nd^ Chance responsible solely for data collection. Data were discussed between the 2 investigators, and a consensus was reached for each event. The definition of critical events was predetermined before the study.

### Sample Size

Based on existing literature and our own experience, we formulated the initial hypothesis, suggesting that the incidence of anesthesia-related critical incidents is at least 3 times higher in resource-limited settings compared to those in well-resourced institutions.^3,18^ Habre et al report an incidence of 3.1% for respiratory critical events, while Cronje et al report an incidence of respiratory critical events of 8.5% in South Africa, around 3 times higher than HICs.^3,18^ Given the specific context of our case series, we opted to perform a sample calculation akin to an observational cross-sectional study. With a confidence level of 95% and a margin of error of 5%, assuming an estimated prevalence of 10%, it was determined, using calculator.net/sample-size, that 138 patients should be included in the study. Considering factors such as the number of patients treated during each mission and the possibility of drop-outs, it was decided to involve patients from 6 consecutive missions, expecting a total of 180 patients to participate in the study.

### Statistical Analysis

Descriptive analyses were performed on the data using Microsoft Excel, Microsoft 365 MSO (Version 2308 Build 16.0.16731.20542). Data related to origin of the patient, ASA status, surgical and anesthesia procedure, and critical events are presented in absolute numbers and percentages. Data related to age, oxygen saturation value, duration of hypoxemia are expressed as the mean and standard deviation (SD).

## RESULTS

### Characteristics of the Patients

Data from 201 patients who underwent surgeries in 4 different hospitals (3 hospitals in DRC and 1 in Tanzania) during 6 missions were collected (Table 2). Among them, 192 patients met the study’s inclusion criteria, while 9 were excluded due to noncompliance with the study protocol). Specifically, during 1 mission in DRC (that was planned to be included in the study), the local team was randomly available, so the anesthesia was not exclusively performed by the local NPAPs. Therefore, all patients from the mission in the Bukavu Hospital in DRC were excluded. Among the included patients, 106 received general anesthesia, while 86 received a combined approach involving both general and regional anesthesia. The general characteristics of the patients are presented in Table 2. All patients from the DRC were adults and underwent thyroid goiter surgery (mean age (±SD) 49(12)) with a female-to-male ratio of 47/2. More than 50% of Tanzanian patients included in the study were between 0 and 5 years old and were ASA I or II. No ASA 3 patients presented in this study. Eighty-six patients had a peripheral nerve block associated with general anesthesia.

### Incidence of Critical Events During General Anesthesia

In the study, 192 patients underwent general anesthesia, and among them, 104 patients (54%) experienced at least 1 event, resulting in a total of 202 events (an average of 1.1 event per patient; Table 3). Specifically, 55 patients (29%) experienced at least 1 episode of hypoxemia, 12 patients (6%) had at least 1 episode of bradycardia, 44 patients (23%) encountered at least 1 episode of airway management problem, and 46 patients (24%) experienced at least 1 equipment problem (see Table 3). Among the episodes of bradycardia, 64% required assistance from AEXP to resolve the issue. Similarly, for airway management and the treatment of hypoxemia, 40% and 64% of incidents, respectively, necessitated the intervention of AEXP.

### Hypoxemia

Hypoxemia affected 55 patients who experienced at least 1 episode, constituting 29% of those who underwent general anesthesia. The median (IQR) age of the patients was 4 (1–27) years. Throughout the study, there were 81 episodes of hypoxemia in total. All of them were successfully managed and 52 of them (64%) were managed with the assistance of AEXP. These incidents happened at different phases, with 31% occurring during induction, 36% during maintenance, and 33% during the awakening phase. Each episode had unique characteristics and causes, and they affected patients of varying ages, origins, and ASA status (Tables 4 and 5). The primary causes of hypoxemia remained the inability to manage the airway properly and intubation failure. Notably, 10% of hypoxemia cases were due to oxygen supply failures (mainly late identification of empty oxygen tank due to barometer malfunction, inability to unscrew the oxygen cylinder regulator due to a worn-out nut and power failure affecting the oxygen concentrator). Patients of all age groups experienced severe hypoxemia (down to 35% oxygen saturation). The longest durations of hypoxemia were observed in the group of patients over 21 years old who were undergoing giant goiter surgery. Among patients between 2 and 5 years old, 34% experienced at least 1 episode of hypoxemia. For children aged 0 to 1, the primary cause of hypoxemia was associated with poor airway management.

### Other Outcomes

Fifty percent of airway management problems led to hypoxemia. No cardiac arrest occurred in the context of the study. All patients were reviewed the day after the procedure without any specific issues. All patients who underwent an axillary block fully recovered without complications. One patient who underwent goiter surgery experienced bleeding that required hematoma drainage, and was not reincluded in the study.

### Missing Data

Several variables specified in the protocol, namely hypo- and hypertension, and tachycardia, could not be measured systematically. The lack of appropriately sized blood pressure cuffs and EKG electrodes resulted in random and incomplete usage of these measurements throughout the study. Since tachycardias cannot be accurately characterized using an oximeter only (precise diagnosis), we decided not to include them in our data collection. Consequently, the occurrence of tachycardia, hypo- and hypertension could not be adequately documented and were excluded from the data collection.

## DISCUSSION

This study provides insights into the occurrence of anesthesia-related incidents in resource-limited settings hospitals in Tanzania and DRC during short-term surgical missions and supports our initial hypothesis that this is substantially higher than for HICs.^18,19^ It reveals that over 50% of the patients experienced at least 1 critical incident. In the majority of cases, the intervention of an expert was necessary to address the issue and avert more serious consequences. The overall incidence of critical events is significantly higher than those reported in relatively similar studies.^3,6,20^

Hypoxemia was the most frequent critical event observed in this study, with 29 % of the patients presenting at least 1 event, similar to that reported by Burgess in Ethiopia (31.3%),^20^ but higher than those reported in limited resource settings by Cronje in South Africa (10.33%),^3^ Ottaway in Namibia (17%),^6^ and Albert in Malawi (17%) (before oximeter training).^21^

If the majority of hypoxemia episodes occur both during induction and awakening regardless of age and ASA class groups, it is clear that, as usual, airway management problems are the primary cause, including intubation failures, endobronchial intubations, mask ventilation difficulties, and accidental extubations.^22^ An essential observation is that in approximately 20% of cases in this study, hypoxemia was the direct result of equipment or oxygen supply failure. For instance, in the 0 to 1 year age group, nearly half of the hypoxemia episodes were attributed to oxygen supply failures, whether stemming from an empty oxygen bottle or a power outage affecting an oxygen concentrator. This illustrates that in both Tanzanian and Congolese hospitals, oxygen supply is a challenge (especially during COVID19 pandemic^23^), and the equipment is so worn out that even when changing the cylinder is anticipated and the tools to change the regulator are ready, one can encounter a completely worn-out bolt that the wrench cannot grip, resulting in several minutes without oxygen delivery.

**Table 5.** Characteristics of the Patients Presenting Hypoxemia

Patient subgroups (number of patient presenting hypoxemia/total)	0–1 y (n = 4/17) (24%)	2–5 y (n = 28/82) (34%)	6–21 y (n = 12/30) (40%)	>21 y (n = 11/63) (18%)	Tanzania (n = 47/143) 33%	DRC (n = 8/49) 16%
Number of hypoxemia events, n	7	44	13	17	67	14
Age, mean (SD)	10 (6) months	3 (1)	8 (3)	43 (14)	5 (6)	50 (10)
ASA status						
I, n (%)	4 (100%)	24 (86%)	9 (75%)	2 (18%)	39 (83%)	0
II, n (%)	0	4 (14%)	3 (25%)	9 (82%)	8 (17%)	8 (100%)
III, n (%)	0	0	0	0	0	0
IV, n (%)	0	0	0	0	0	0
Sex (M/F)	4/0	15/13	7/5	4/7	26/21	4/4
Country						
Tanzania (reconstructive surgery), n (%)	4 (100%)	28 (100%)	12 (100%)	3 (27%)	47 (100%)	0
DRC (giant goiter surgery), n (%)	0	0	0	8 (73%)	0	8 (100%)
Timing of hypoxemia						
Induction, n (%)	2 (29%)	10 (22%)	2 (15%)	11 (64%)	14 (21%)	11 (79%)
Maintenance, n (%)	5 (71%)	18 (41%)	2 (15%)	3 (18%)	25 (37%)	3 (21%)
Emergence, n (%)	0	16 (37%)	9 (70%)	3 (18%)	28 (42%)	0
Duration of hypoxemia in seconds, mean (SD)	60 (57)	97 (119)	77 (85)	158 (127)	88 (106)	175 (132)
Lowest pulse oximeter value (%), mean (SD)	72 (18)	76 (15)	76 (11)	69 (17)	76 (14)	67 (17)
Minimal pulse oximeter value during hypoxemia	43	35	54	40	35	40
Maximal pulse oximeter value during hypoxemia	90	90	88	90	90	90
Cause of hypoxemia						
Airway management failure, n (%)	4 (57%)	11 (25%)	2 (15%)	8 (47%)	17 (25%)	8 (57%)
○ Intubation failure, n	1	2	1	7	4	7
○ Endobronchial intubation, n	2	5	1	1	8	1
○ Facemask ventilation failure, n	0	2	0	0	2	0
○ Accidental extubation, n	1	2	0	0	3	0
Bronchospasm, n (%)	0	13 (30%)	2 (15%)	0	15 (22%)	0
Hypoventilation, n (%)	0	10 (23%)	2 (15%)	1 (7%)	13 (20%)	0
Laryngospasm, n (%)	0	4 (9%)	5 (40%)	0	9 (14%)	0
Anesthesia machine failure, n (%)	0	2 (4%)	0	4 (23%)	2 (3%)	4 (29%)
Oxygen supply failure, n (%)	3 (43%)	3 (7%)	0	4 (23%)	8 (12%)	2 (14%)
Aspiration, n (%)	0	1 (2%)	0	0	1 (1%)	0
Other, n (%)	0	0	2 (15%)	0	2 (3%)	0

n indicates total number, and % indicates proportion of total cases (n).

Abbreviation: ASA, American Society of Anesthesiologists; DRC, Democratic Republic of the Congo; SD, standard deviation.

These observations illustrate the multifactorial nature of the occurrence of a critical event like anesthesia-related hypoxemia in resource-limited conditions. One might also wonder why the overall rate of critical events, and hypoxemia in particular, is so high compared to what is known in sub-Saharan^3,6,21^ or in low- and middle-income countries.^24^ One possible explanation could be linked to the lack of resources in rural areas compared to large urban centers.^25,26^ On one hand, it is indeed well-known in the literature that equipment and anesthesia drug resources are desperately insufficient in rural areas.^27^ On the other hand, anesthesia in these regions is often administered by NPAPs with varying levels of training, frequently using ketamine for general anesthesia which allows spontaneous breathing without advanced airway management or as a replacement for certain techniques like spinal anesthesia.^28^

### Study Limitations

This study is conducted as part of a mission, either in thyroid surgery in the DRC or in reconstructive surgery in Tanzania, which can, on one hand, destabilize a local team accustomed to a lower workload, simpler anesthesia without intubation, unfamiliar patients and pathologies, and on the other hand, contribute to a more significant and acute consumption of resources (electricity, oxygen, medications, equipment). It may appear unwise to evaluate adverse events within teams that have less experience with certain types of patients. However, in Tanzania, the teams regularly handle pediatric patients undergoing reconstructive surgeries. Regarding the DRC, a previous study showed no significant difficulties in managing the airways of patients with giant goiters (98% of patients had a Cormack-Lehane grade of 1 or 2),^29^ and the local teams reported to be experienced in administering general anesthesia to adult patients for other types of surgeries. Nevertheless, the specific context of the surgical mission study may have overestimated the number of critical events given the unusual nature of the surgery. This is particularly true in DRC where giant goiter surgery was not previously performed in these hospitals. It is undoubtedly interesting to note that the hypoxemia cases in the DRC group for goiter surgery were due to intubation failures and equipment and supply shortages. Even though giant goiters are not a known factor for difficult intubation in Eastern DRC for an anesthesiologist,^29^ it is highly likely that this condition contributes to intubation difficulties. The local NPAP teams in both Tanzania and the DRC were heterogeneous in terms of skills and experiences. Although we did not measure it, it is possible that the least experienced NPAPs faced more critical events.^6^ It is also possible that the anesthetic strategy selected by the NPAP would have been different without the presence of the AEXP. Moreover, due to the lack of appropriate equipment, we faced a deviation from the protocol and decided not to collect data on tachycardia, hypotension and hypertension. Bradycardia was prioritized in monitoring and documentation due to its significant clinical implications, particularly in the context of using halothane with succinylcholine in pediatric patients.^30^

Current efforts focus on empowering NPAPs to advocate for permanent solutions within their hospitals, while also acknowledging the potential benefits of introducing basic monitoring equipment during mission trips to further reinforce the importance of these tools and support sustainable change. Additionally, the total number of incidents may be overestimated in this study, as we count hypoxemia, airway management issues, and oxygen supply and equipment failures as independent critical events when they are clearly interconnected, and hypoxemia is just the clinical sign that results from them. Finally, these results are likely not generalizable to an entire region or to all hospitals, as there may be significant differences in terms of equipment and human resources among them.

In conclusion, this study highlights that anesthesia in resource-limited settings presents challenges and risks. The incidence of critical events appears to be higher in this study compared to what is reported in HICs. The most common event is hypoxemia, which can occur at any point during anesthesia and affects all age and risk groups. Difficulties in airway management and problems with equipment and oxygen supply are the most frequent causes. One potential solution to this issue is to enhance the knowledge and abilities of NPAPs, increase the recognition of their contributions by surgeons, and improve the equipment and monitoring resources available. Documenting this high incidence of critical events related to anesthesia in resource-limited settings will contribute to advocating for the recognition of the often-neglected needs in anesthesia. The hope is that the minimal standards defined by the WFSA-WHO will be recognized and implemented in the real lives of surgical teams, not just in political rhetoric. Improving resources with equipment that meets international standards and is adapted to local conditions, along with specialized training in airway management and hypoxemia, should reduce the incidence of these critical events. Further studies are needed to evaluate the impact of enhanced equipment and training on clinical outcomes, particularly in reducing hypoxemia. Future surgical missions should ensure that the clinical practice meets the minimum standards such as the WHO-WFSA standards of anesthesia practice. This may be achieved by bringing basic monitoring equipment (oximeters, adapted blood pressure cuffs, EKG, and capnography) by the visiting team and conducting formal advocacy to the hospital administration about the availability of equipment. In addition, local NPAPs team should be trained on management of new surgical cases.

## ACKNOWLEDGMENTS

We would like to thank all the anesthesia team from “2^nd^ Chance” who participated in data collection: Mr Jean-Daniel Junod (nurse anesthetist, Geneva University Hospital, Switzerland), Dr Michel Pellegrini (anesthesiologist, Geneva University Hospital, Switzerland), Dr Bertrand Gautier (anesthesiologist, Geneva University Hospital, Switzerland), Dr Andres Hagerman, MD (anesthesiologist, Geneva University Hospital, Switzerland), Dr Iris Pelieu (anesthesiologist, Geneva University Hospital, Switzerland), Dr David Grosser, MD (anesthesiologist, Kaiser Permanente, Denver, CO), Dr Krystofer Lysakowski, MD (anesthesiologist, Geneva University Hospital, Switzerland), Dr David Rodriguez, MD (anesthesiologist, Hôpitaux Privés de Savoie, France), and Dr Alexandre Cereuil (Marseille University Hospital, France). Paper Reviewing: Dr Laszlo Vutskits, MD, PhD (anesthesiologist, Geneva University Hospital, Switzerland). Financial support of the 2^nd^ Chance anesthesia project: Fondation d’Anesthésie Réanimation (FAR); City and Canton of Geneva.

## DISCLOSURES

**Conflicts of Interest:** None. **Funding:** None. **This manuscript was handled by:** Angela Enright, MB, FRCPC.

## Supplementary Material


