# Supplementary material for: Intraoperative Anesthesia-Related Critical Events in Low-Resource Hospitals During Short-Term Surgical Missions in Tanzania and Democratic Republic of the Congo: An Observational Study
Source: Anesth Analg. 2024 Oct 28;140(3):646–54. doi: 10.1213/ANE.0000000000007317 (PMC11805488; doi:10.1213/ANE.0000000000007317)
Supplement: Supplementary file 1 [file ane-140-646-s001.docx]

**Anesthesia-related critical events study in the DRC and Tanzania**

1. **General information** (please write or circle the correct option)

| - Place of mission |  |
| --- | --- |
| - 2^nd^ Chance anesthesiologist / local |  |
| - Date |  |
| - Patient’s code number |  |
| - ASA | 1 2 3 4 |
| - Gender | M / F |
| - Age |  |
| - Weight |  |
| - Type of surgery |  |
| - Duration of surgery |  |
| - Type of anesthesia | General / Regional / Combined (RA + GA) / Spinal |

1. **Critical Events** (please write or circle the correct option)

**2a) Hypoxemia**

*Definition : SpO2 <90% for any length of time*

| - Hypoxemia (at least one event) | YES / NO |
| --- | --- |
| - Number of events |  |
| **EVENT NUMBER 1** | |
| - Lowest SpO2 value |  |
| - Timing of hypoxemia | Induction / Maintenance / Emergence |
| - Cause of hypoxemia | - Airway Management failure (describe) - Bronchospasm - Laryngospasm - Hypoventilation - Anesthesia machine problem - Oxygen supply failure - Aspiration - Other (describe) |
| - Duration of hypoxemia (in seconds) |  |
| - Management (of local team) | SELF / WITH HELP |
| **EVENT NUMBER 2** | |
| - Lowest SpO2 value |  |
| - Timing of hypoxemia | Induction / Maintenance / Emergence |
| - Cause of hypoxemia | - Airway Management failure (describe) - Bronchospasm - Laryngospasm - Hypoventilation - Anesthesia machine problem - Oxygen supply failure - Aspiration - Other (describe) |
| - Duration of hypoxemia (in seconds) |  |
| - Management (of local team) | SELF / WITH HELP |

| **EVENT NUMBER 3** | |
| --- | --- |
| - Lowest SpO2 value |  |
| - Timing of hypoxemia | Induction / Maintenance / Emergence |
| - Cause of hypoxemia | - Airway Management failure (describe) - Bronchospasm - Laryngospasm - Hypoventilation - Anesthesia machine problem - Oxygen supply failure - Aspiration - Other (describe) |
| - Duration of hypoxemia (in seconds) |  |
| - Management (of local team) | SELF / WITH HELP |

**NB :**  If more than 3 hypoxemia events, please recreate more tabs.

**2b) Bradycardia**

*Definition: HR <50 bpm (adults) ; Age-adjusted in children (referring table)*

| - Bradycardia | YES / NO |
| --- | --- |
| - Number of events |  |
| **EVENT NUMBER 1** | |
| - Lowest HR |  |
| - Management (of local team) | SELF / WITH HELP |
| - Use of Atropine | YES / NO |
| **EVENT NUMBER 2** | |
| - Lowest HR |  |
| - Management (of local team) | SELF / WITH HELP |
| - Use of Atropine | YES / NO |

**NB :** If more than 2 bradycardia events, please recreate more tabs.

**2c) Tachycardia**

*Definition: HR>100/min ; Age-adjusted in children (referring table)*

*NB : EKG is necessary in order to have the correct diagnosis.*

| - Tachycardia | YES / NO |
| --- | --- |
| - Number of events |  |
| - Sinus Tachycardia | YES / NO |
| - If No : | Supraventricular / Ventricular / Unknown |
| - Treatment or action to correct : |  |
| - Management (of local team) : | SELF / WITH HELP |

**NB :** If more than 1 event, please recreate more tabs.

**2d) Hypotension**

*Definition: Systolic <90 and/or Diastolic <60 (Adults) ; ; Age-adjusted in children (referring table)*

| - Hypotension | YES / NO |
| --- | --- |
| - Number of events |  |
| - Lowest value (Systolic / Diastolic / MAP) | S : / D : / MAP: |
| - Identified cause : |  |
| - Treatment or action to correct : |  |
| - Management of local team : | SELF / WITH HELP |

**NB :** If more than 1 event, please recreate more tabs.

**2e) Hypertension**

*Definition: Systolic >140 and/or Diastolic >90 (Adults) ; ; Age-adjusted in children (referring table)*

| - Hypertension | YES / NO |
| --- | --- |
| - Number of events |  |
| - Highest value (Systolic / Diastolic / MAP) | S : / D : / MAP: |
| - Identified cause : |  |
| - Treatment or action to correct : |  |
| - Management of local team : | SELF / WITH HELP |

**NB :** If more than 1 event, please recreate more tabs.

**2f) Airway Management problems**

| - Number of events |  |
| --- | --- |
| - Face mask ventilation failure |  |
| - > 2 laryngoscopies | YES / NO |
| - Selective intubation | YES / NO |
| - Incidental extubation | YES / NO |
| - Esophageal intubation | YES / NO |
| - Other (please describe) |  |
| - Management (of local team) | SELF / WITH HELP |

**2g) Equipment incidents**

| - Medication failure or incident | YES / NO |
| --- | --- |
| - If YES, please describe (free text) |  |
| - Material/Equipment incident | YES / NO |
| - If YES, please describe (free text) |  |
